# Characterization of the complete mitochondrial genome of the fork-tailed threadfin bream, *Nemipterus furcosus* (Spariformes, Nemipteridae) and phylogenetic analysis

**DOI:** 10.1080/23802359.2020.1778576

**Published:** 2020-08-07

**Authors:** Ha Yeun Song, Yun-Hwan Jung, Young Ji Choi, Bora Kim, Tu Van Nguyen, Dae-Sung Lee

**Affiliations:** a Department of Genetic Resources Research, National Marine Biodiversity Institute of Korea, Seocheon-gun, Republic of Korea; b International Center for Marine Biodiversity, National Marine Biodiversity Institute of Korea, Seocheon-gun, Republic of Korea; c Department of Ecology, Institute of Tropical Biology, Vietnam Academy of Science and Technology, Ho Chi Minh City, Vietnam

**Keywords:** Mitochondrial genome, Spariformes, Nemipteridae, *Nemipterus furcosus*

## Abstract

The complete mitochondrial genome of the fork-tailed threadfin bream, *Nemipterus furcosus*, which belongs to the family Nemipteridae was first determined. The complete mitochondrial genome was 16,882 bp in size and encoded of 13 protein-coding genes, 22 tRNA genes, 2 rRNA genes, and a control region. *Nemipterus furcosus* has a mitochondrial gene arrangement that is typical of vertebrates. Phylogenetic analysis using mitochondrial genomes of 11 related species revealed that *N. furcosus* formed a well-supported monophyletic group with the other Nemipteridae species. This mitochondrial genome provides a useful information for addressing taxonomic issues.

The fork-tailed threadfin bream, *Nemipterus furcosus* (Spariformes, Nemipteridae), is a tropical reef-associated marine fish widely distributed in the West Pacific from southern Japan to northeastern Australia, and Indian Ocean including the Gulf of Mannar, Sri Lanka, Andaman Sea, Strait of Malacca and northwestern Australia (Russell 1990). The placement of the Nemipteridae and associated families (Sparidae and Lethrinidae) has been controversial in the order Spariformes. Although molecular and morphological studies reported the Nemipteridae belong to the order Spariformes (Johnson 1981; Carpenter and Johnson 2002; Sanciangco et al. 2016), the position of this family is unclear. Here, we first determined the complete mitochondrial genome sequence of *N. furcosus* and analyzed the phylogenetic relationship of this species with members of Nemipteridae and Sparidae.

The *N. furcosus* specimen was collected from Ho Chi Minh City, Vietnam (10.53 N, 106.45 W). Total genomic DNA was extracted from the specimen tissue, which has been deposited at the National Marine Biodiversity Institute of Korea (Voucher No. MABIK0002423). The mitogenome was sequenced using Illumina Hiseq 4000 sequencing platform (Illumina, San Diego, CA) and assembled with *SOAPdenovo* at Macrogen Inc. (Seoul, Korea). The complete mitochondrial genome was annotated using MacClade ver. 4.08 (http://macclade.org/macclade) (Maddison and Maddison 2005) and tRNAscan-SE ver. 2.0 (http://lowelab.ucsc.edu/tRNAscan-SE) (Lowe and Chan 2016).

The complete mitochondrial genome of *N. furcosus* (GenBank accession no. LC549804) is 16,882 bp in length and includes 13 protein-coding genes, 22 tRNA genes, 2 rRNA genes, and a control region. The overall base composition is 28.14% A, 28.46% C, 16.79% G, and 26.61% T. Similar to the mitogenomes of other vertebrates, the AT content is higher than the GC content (Saccone et al. 1999). All tRNA genes can fold into a typical cloverleaf structure, with lengths ranging from 66 to 74 bp. The *12S rRNA* (1000 bp) and *16S rRNA* genes (1758 bp) are located between tRNA^Phe^ and tRNA^Val^ and between tRNA^Val^ and tRNA^Leu(UUR)^, respectively. Of the 13 protein-coding genes, 12 start with ATG; the exception being *COI*, which starts with GTG. The stop codon of the protein-coding genes is TAA (*ND1*, *COI*, *ATP8* and *ND6*), T (*COII*, *ND3*, *ND4* and *Cytb*), TA (*ND2*, *ATP6* and *COIII*), TAG (*ND4L*) and AGA (*ND5*). A control region (1096 bp) is located between tRNA^Pro^ and tRNA^Phe^.

The phylogenetic trees were constructed by the maximum-likelihood method using MEGA 7.0 software (MEGA, Philadelphia, PA) (Kumar et al. 2016). We analyzed the phylogenetic trees of the newly sequenced genome and 11 other complete Nemipteridae and Sparidae species mitochondrial genome sequences acquired from the National Center for Biotechnology Information. We confirmed that *N. furcosus* formed a monophyletic group with the other Nemipteridae species (Figure 1). This mitochondrial genome provides an important resource for addressing taxonomic issues.

**Figure 1.**
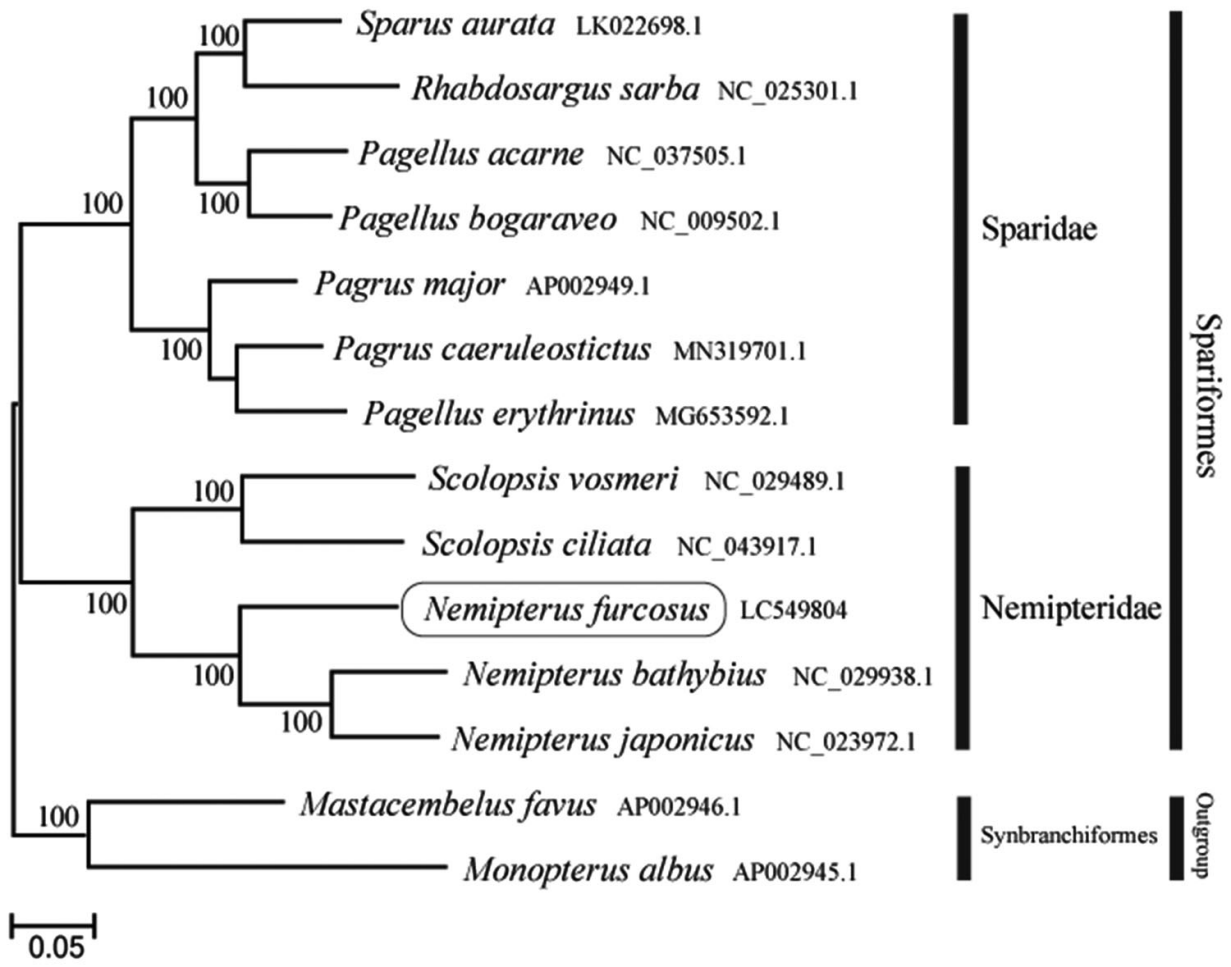
Phylogenetic position of *Nemipterus furcosus* based on a comparison with the complete mitochondrial genome sequences of 11 related species. The analysis was performed using MEGA 7.0 software. The accession number for each species is indicated after the scientific name.

## Data Availability

The data that support the findings of this study are openly available in the DNA Data Bank of Japan (accession no. LC549804) at https://www.ddbj.nig.ac.jp.
